# Supplementary material for: Phosphatidyl Ethanolamine Binding Protein FLOWERING LOCUS T-like 12 (OsFTL12) Regulates the Rice Heading Date under Different Day-Length Conditions
Source: Int J Mol Sci. 2024 Jan 24;25(3):1449. doi: 10.3390/ijms25031449 (PMC10855395; doi:10.3390/ijms25031449)
Supplement: Supplementary file 1 [file ijms-25-01449-s001.zip › OsFTL12 SP Table/OsFTL12 SP file proof.pdf]

1 ATGGCAAATGACTCATTGACAAGGAGCCATATAGTTGGAGATGTG 46 TTAGACCAATTTTCAAACTCAGTGCCTCTAACTGTGATGTATGAT

M A N D S L T R S H I V G D V L D Q F S N S V P L T V M Y D

91 GGGAGGCCTGTGTTTAATGGCAAGGAGTTCCGTTCCTCGGCAGTC 136 TCGATGAAACCTAGAGTTGAGATTGGTGGCGATGATTTTCGATTT

G R P V F N G K E F R S S A V S M K P R V E I G G D D F R F

181 GCCTATACCCTAGTTATGGTGGATCCTGATGCTCCTAATCCCAGC 226 AACCCAACCTTGAGGGAATACCTGCACTGGATGGTGACTGATATC

A Y T L V M V D P D A P N P S N P T L R E Y L H W M V T D I

271 CCATCATCGACGGACGATAGCTTTGGGCGGGAGATCGTAACATAC 316 GAAAGCCCAAGCCCCACCATGGGCATCCACCGCATCGTGATGGTG

P S S T D D S F G R E I V T Y E S P S P T M G I H R I V M V

361 TTGTATCAGCAGCTTGGGCGCGGCACGGTGTTCGCGCCGCAGGTG 406 CGTCAGAACTTCAACCTGCGCAGCTTCGCGCGCCGTTTCAACCTC

L Y Q Q L G R G T V F A P Q V R Q N F N L R S F A R R F N L

451 GGCAAGCCGGTGGCCGCCATGTACTTCAACTGCCAGCGCCCGACA 496 GGCACAGGTGGGAGGAGGCCAACCTGA

G K P V A A M Y F N C Q R P T G T G G R R P T *

Figure S1. *OsFTL12* CDS sequence and translated amino acid sequence.

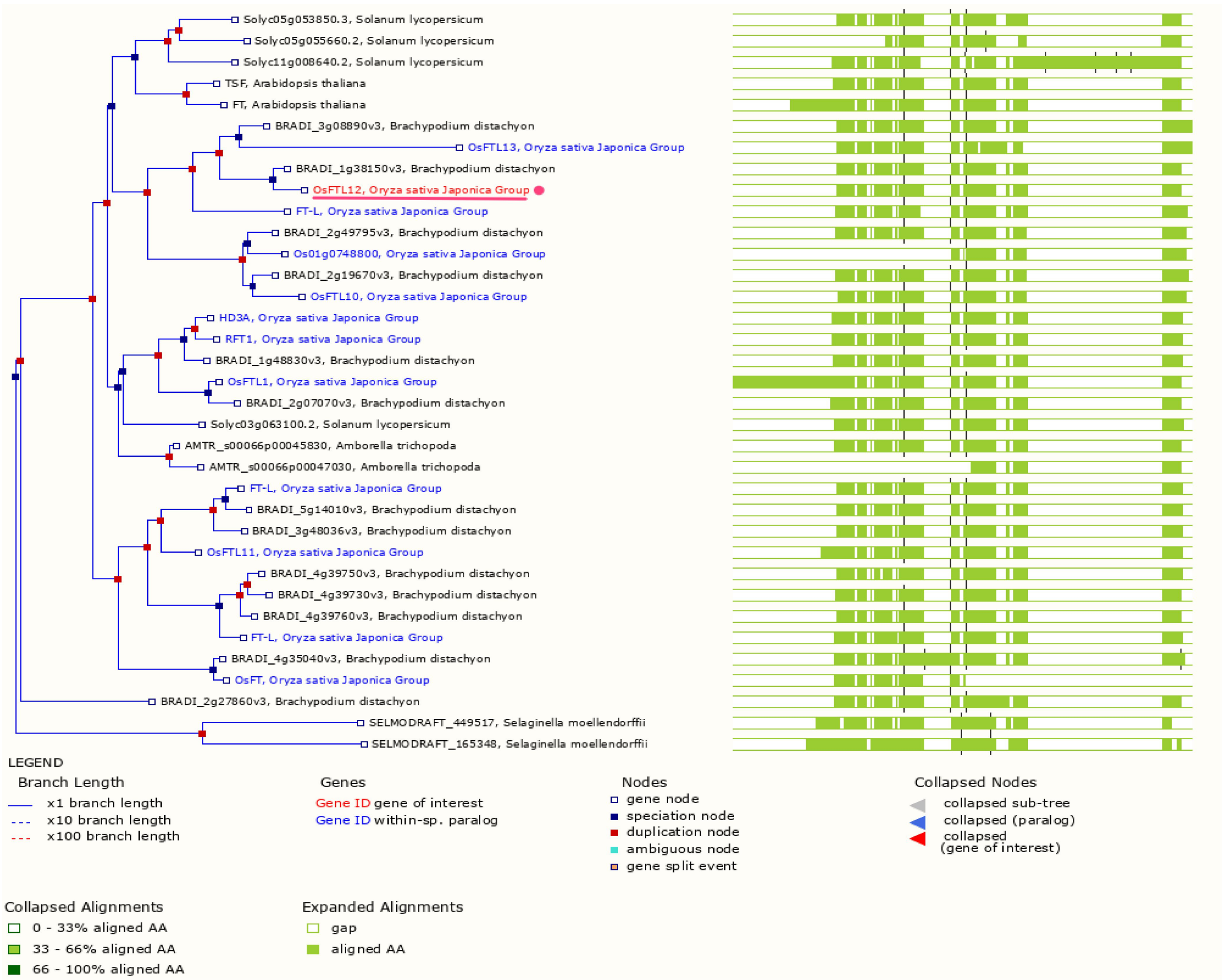

Figure S2. Phylogentic tree of *OsFTL12* orthologues in plant species. Photograph generated by blast the *OsFTL12* in Ensembl Plants database.

5’-GGGACAGACGACAATTTTTTTCTAAAATTCACTCACATGGGGAGTCGAACTCAAGACTTGA
GAGTGCTACTAGAGCCACCTAACCAACTCAGCTAGAGGCCCGTTCGCTAGCGTCAGACATATA
GCCTCAACCACTCAGACTAGCCGTTTGAGCCTGGTCAGTAGTTCAAAGTTCACAGTGTGACAA
CAATCGAAGAAGTATTGACCTGGTTTCTCCGGTACAACCCGGTCCTTGCGGATCACCGTTATTC
TGGACTGTCTTGTAGCTCTCAGACTATCTGAGCAATGCAACTCCACAATAAGTGCGAACATATT
GCACACTATATGAGTTACACACATCGGACTGTCATCCATTTTATTTCCACTATACGGCCAACACA
TGGCCCATTTGGACAATGAATTGATCGTAATGTCCGATCCAATGGTTTAGCCGGACTGTCTACC
ATTACTGGGACTGTCCAGCTAGTTCAACTTTCAGGGTAACATCAAAGATCTTTGTTTGCAGCAT
ATGCCAACACTCCCACTTCATTTAGTATTTGATTTGAAAATATATAGCAAGCTAGTGTACACCAC
TCCTAGACTCCTTGCAACTCCAGGCTACTTAAATTTCATCATATCTTTATAGTACGGTCTTTTGGT
TCTAAAACTATTGCATTCACCCAACTCAAACACAAAGGTTCCACAATTCGATTTAATTTACCGC
ACCATAACTCACAAGGGGATACACCAAACAATTTTGCATCATGCATGACTCCATAAAGTCCAAC
AGAACCCTAATCTTTGCACACTTAGCAACTATCACTACTATAGAAAATGCTTTAGCCTTTAGGTT
TGATTGCTAGCCCCACTTGAGTACCGGATTTGACAATCGGCACCCAGCAGGCAACACCTTTGA
GTTCTCTCAAAGGTGTCGGTTTAAGAACTGGCACCTTTAAGGTCCAAGGAATGAAAAGCAAA
GGTCGGCTCAATTCGAGCTGATGCATCGAGCAACTTCCCTTTACCCCAATCAGATCTCATTGCA
CATTTAGTTGCATTTAATCCAAGAAAAGGCAAGAACACTAAAAACATAAGAACATCAACAACA
CAAGCAAGGGGAGGGGATGGGAGGGTGTCGGCCGCCATAGCCGCCTCCCCTTCCACTGGAT
CTAGCGGAGGGGAGGTCGCTGCCGCCGCTGGGTGGGAGCAGTGGAGAGAGAGGGGGACC
GAGAGAGAGAGCAGTGGATAGAGGAGGAGATAGAGGGAGGAGATAAGGAGGAGAGAGA
GAGAGGTGGGTGGGTGCGTTGACGAGATAATTAAGGCCGAGGGGAGAGCGCGCGGGTCAG
GCTATGCTTGATTTTTCAGGTGTCGTTTTTTTAAAAAAAATCGACACCTATATAATTGGTGGCG
GTTTTTCTTTACAACCGGCACCTATAATTACTTAAAGGCATCAGTTCTATATATTTTCAGATCGTA
GAGGTAGGAAAAAAAATCTATAGGTACTGGTTTTAATACTTCCGGCAACTATAGTGTTGACTTC
TATGTGTATTTTTTTTGTAGTAGTGTATGATTAGTTCCGATGATTATGTTGTTATTAAGCGCTAAA
ATAACCATAAAGGGGCCTAGATGCTTGCACCCAGTAGAAATAGTGTATCGAGCATTCCAGTCG
ATCGACGCACTCCTCCAATCGGCCAATCCCACAACCCCGATCCATGGGAAAGGAAAGAGCCA
AATTACCATGACACTATGGAACATGATGCATTAAAGATGCTACAAAATTAATTGTATATAACTGAT
ATGGCACAGATATGTTTATTCCCAGACATCCGCCATGCTGCAAATTGAAATAGCAAATGGAAGA
ATAATGTGTATAATCCTGTCAAACAATCAGAAGAGCATCTTGTAGAAAGTAGATTCAATACTATT
GAAATTGATAAAACGGGAATCACATGTGTGCGCAATGAGACAGCATCCTGTCGTGTAGACCAC
TATAAATAGCAGCAAAACCTAGTCTGTTTTTCCCA-3’

Figure S3. *OsFTL12* promotor sequence. The promotor located at 2kb upstream of *OsFTL12* 5’-UTR sequence.

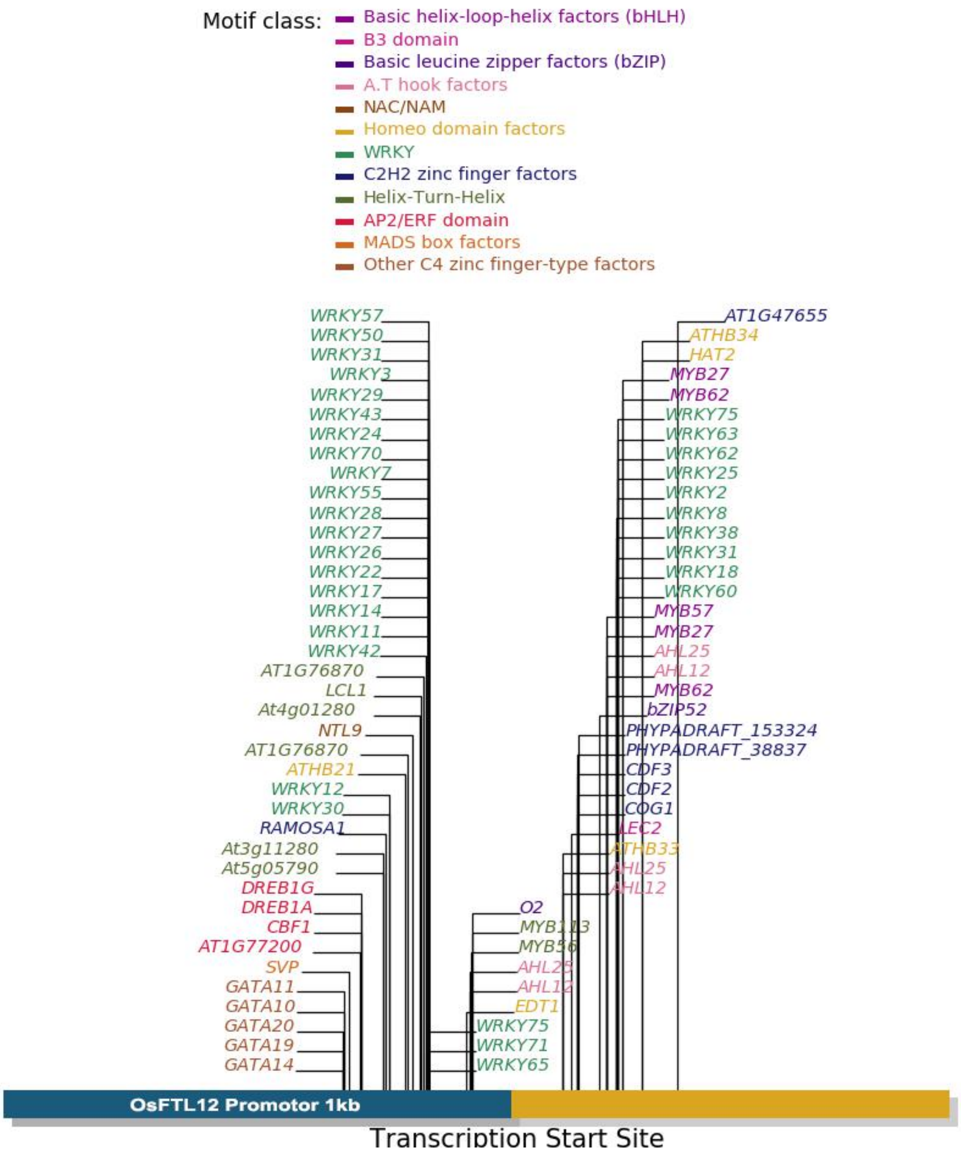

Figure S4. Plant regulomics database predicted putative *Arabidopsis* transcription factors binding sites in *OsFTL12* promotor sequence (1kb).

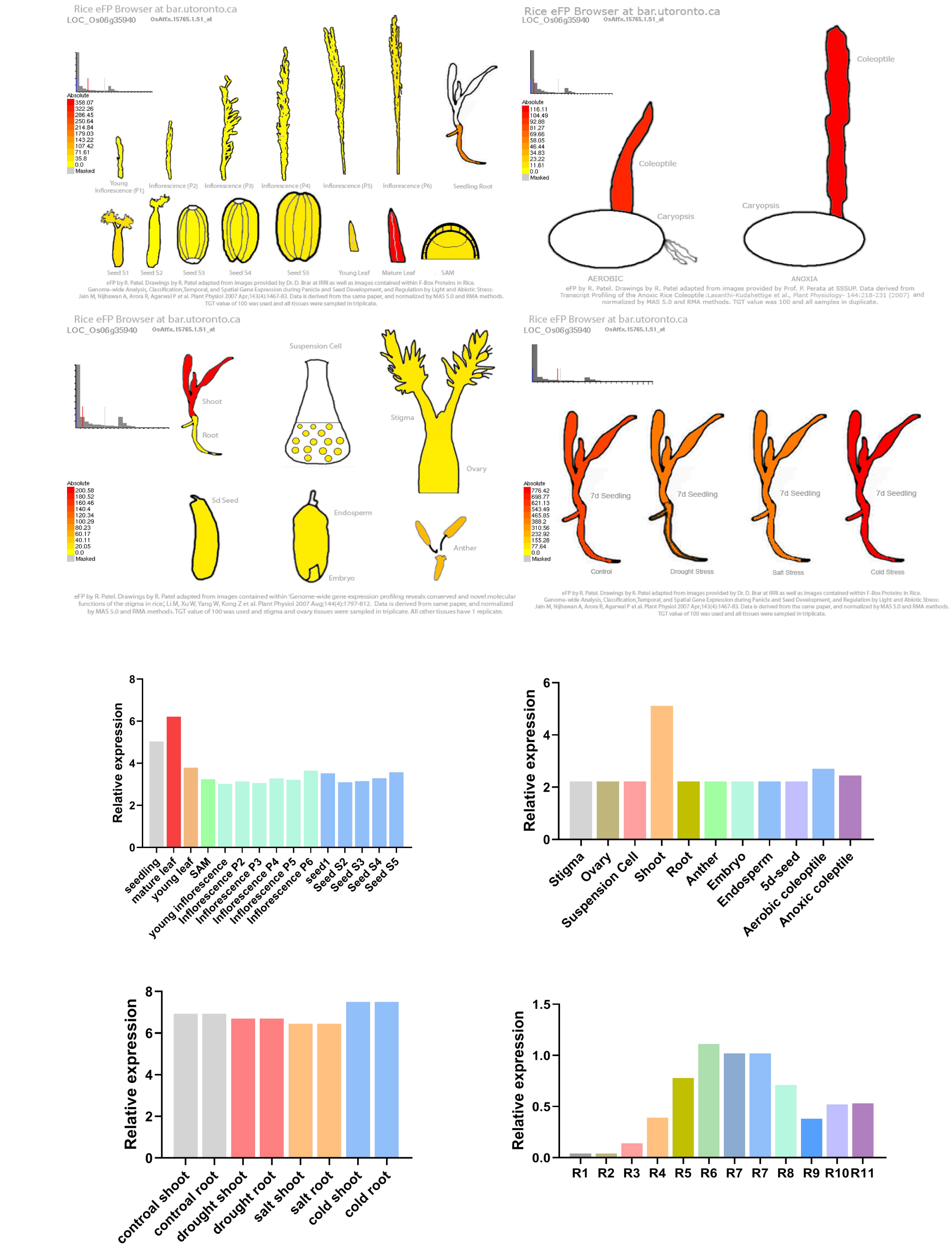

Figure S5. Expression values of *OsFTL12* in different tissues. This results were provided by Electronic Fluorescent Pictograph Data-sets published data.

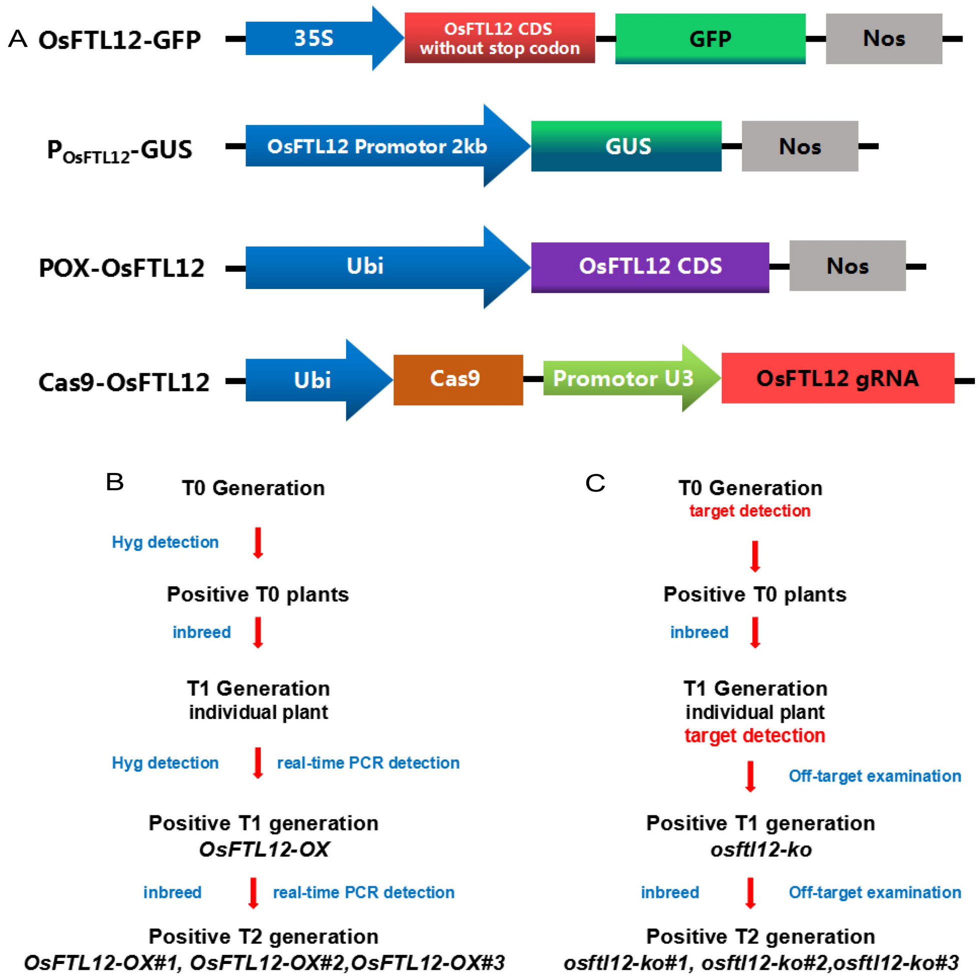

Figure S6. Generated procedure of *OsFTL12* transgenic plants. A, plasmid construction for subcellular localization analysis, GUS staining assay, *OsFTL12* over-expressing plant, and CRISPR/Cas9 induced *osftl12-ko* plant. B, screening procedure of *OsFTL12-OX* plants. C, Detail procedure of obtained the *osftl12-ko* plants.

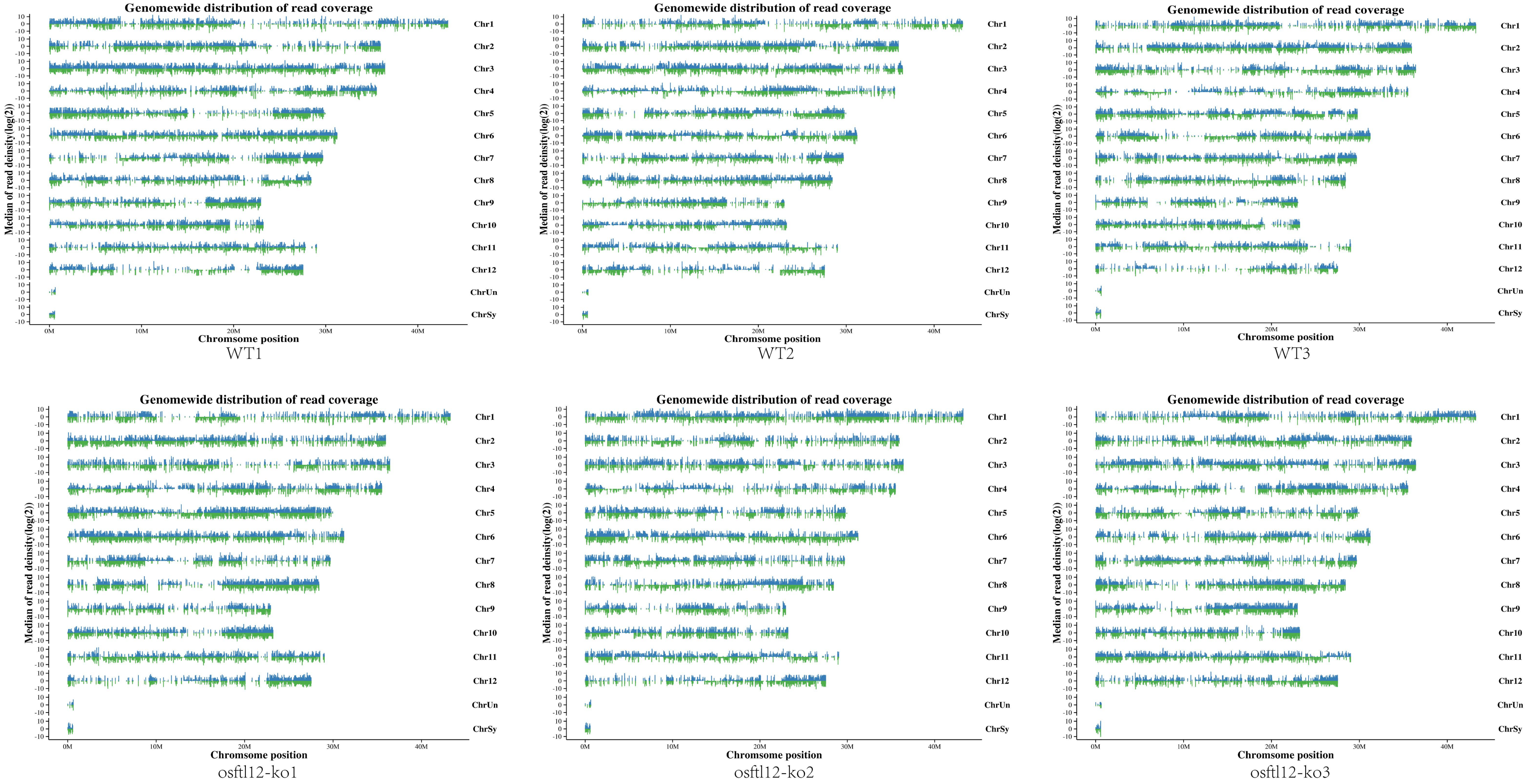

Figure S7. Raw RNA-seq reads mapped ration in each sample.

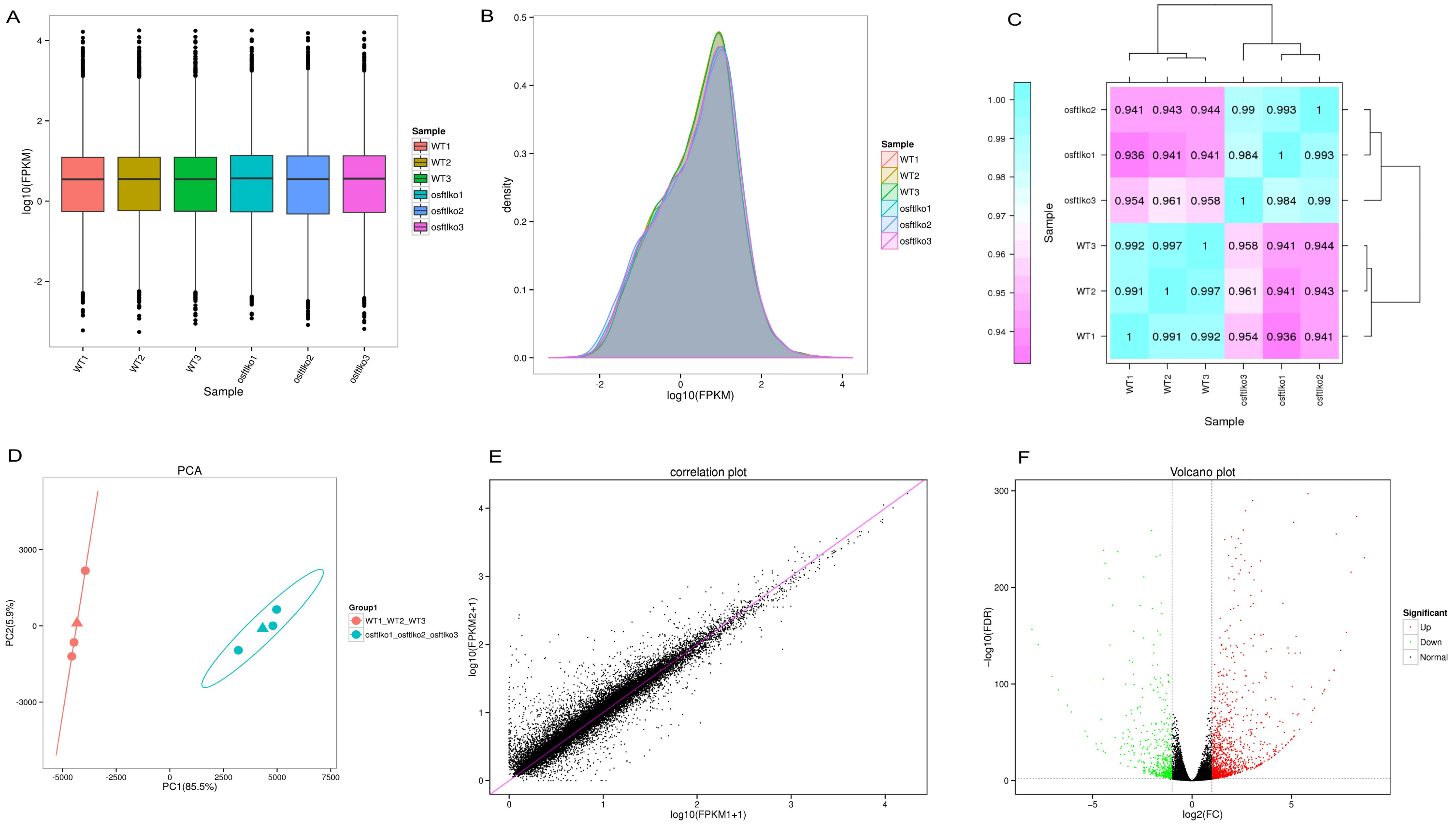

Figure S8. Brief data processing of DEGs identification. A, All gene FPKM expression box. B, All gene FPKM expression density. C, sample correlation. D, PCA analysis of all sample. E, Dorrelation plot of all DEGs. F, Volcano plot of DEGs screening parameter, $log^2FC > 1$.

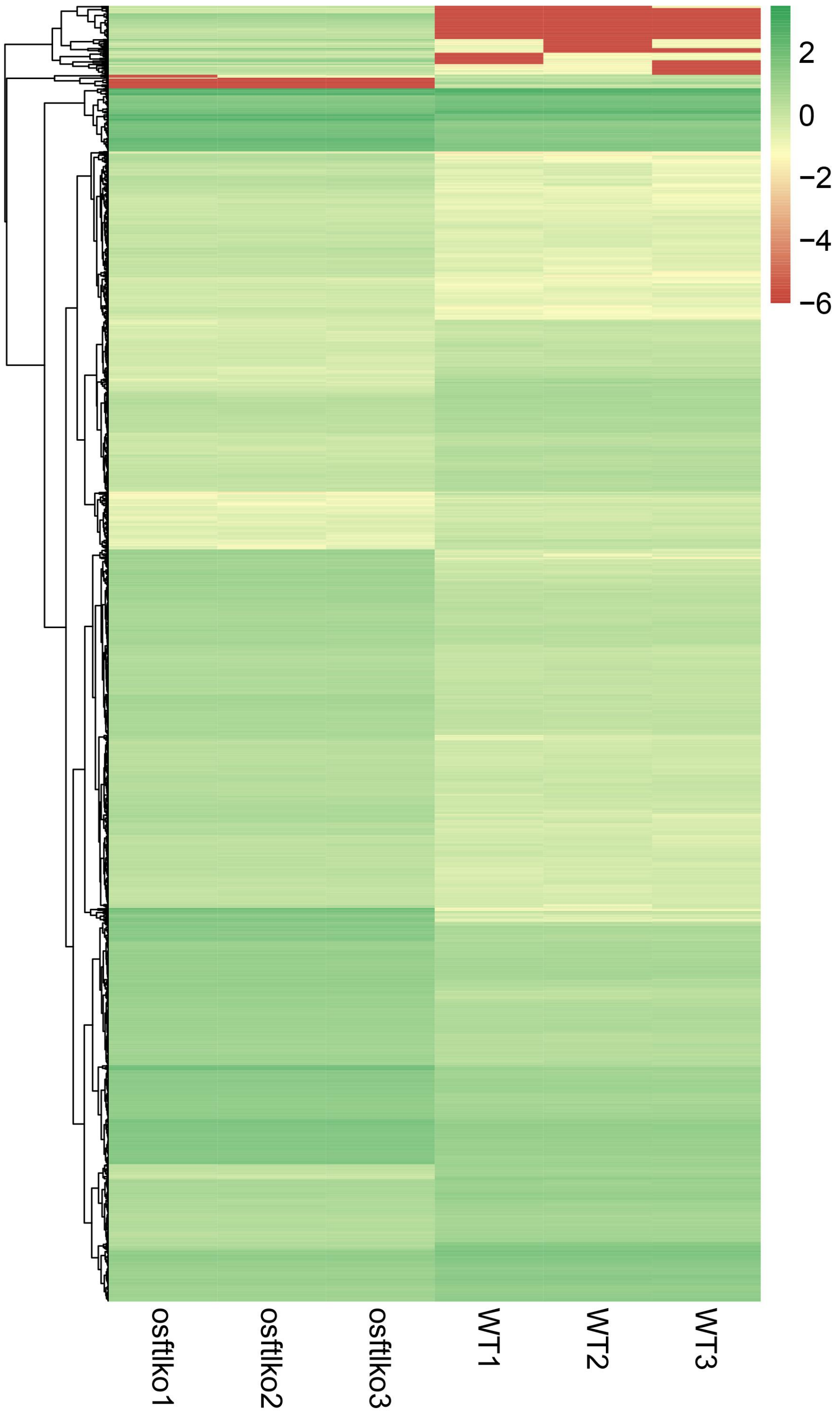

Figure S9. Heatmap displayed the DEGs expression level in each sample.

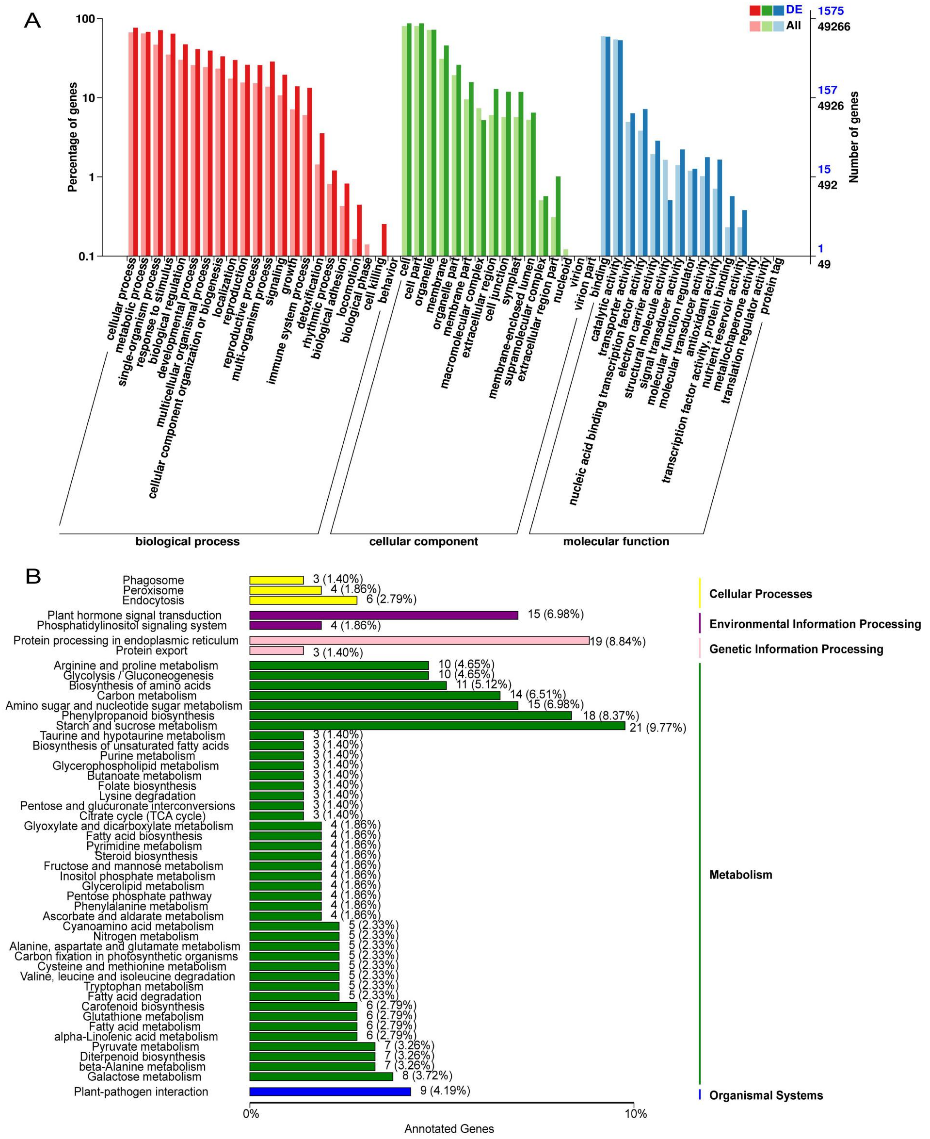

Figure S10. GO analysis and KEGG enrichment of DEGs in the transcriptome profile of *osftl12-ko* vs. WT. A-B; GO and KEGG analysis of all RNA-seq identified DEGs in *osftl12-ko* profile, respectively.
